# Improving weight loss RCTs. Measuring the step weight change from a sustained change in frequency of a particular eating or exercise pattern

**DOI:** 10.1038/s41430-022-01247-7

**Published:** 2022-12-06

**Authors:** David A. Booth, Antonio Laguna-Camacho

**Affiliations:** 1 School of Psychology, University of Sussex, Falmer, Brighton, UK; 2 School of Medicine, Autonomous University of the State of Mexico, Toluca, Mexico

**Keywords:** Weight management, Education

## Abstract

The public’s trust in the science of avoiding unhealthy weight depends on a radical reform of the design and execution of weight loss programmes and their clinical trials. This Perspective reiterates the longstanding argument for measuring the effectiveness of each component of an intervention on obesity. Body energy content change results from a difference in rates between input and output. These rates are determined by the frequencies of specific patterns of dietary behaviour, physical activity and thermal comfort, plus the cost of resting metabolism. Since fat-free mass changes alongside fat mass, the amount of change in weight from a change in the frequency of a behaviour pattern comes to an asymptote. That step change in weight per unit of behaviour change is measured by regression from the change in frequency of the behaviour that has been maintained from baseline to follow-up. For hard evidence, weight loss programme participants’ own words must be used to specify behaviour. In RCTs of multiple-component programmes, sequences of the behaviour patterns to be changed are randomised among groups. The resulting evidence on effective slimming practices can be delivered directly into therapeutic services and public health interventions for the culture investigated.

The design and execution of weight loss programmes and their clinical trials need radical reform. Morbidity and mortality associated with the high prevalence of obesity must be reduced more effectively than to date [1, 2]. The long-term trust of the public in therapeutic services and public health interventions is at risk [3]. This Perspective reiterates the longstanding argument for measuring the effectiveness of each component of an obesity reduction programme [4, 5]. New insight comes from the first example of a key measurement approach based on a shift in the usual number of episodes per week of a locally specified pattern of behaviour that influences the rate of intake or expenditure [6].

Such measurements can refute two mistaken assumptions that undermine efforts to reduce obesity. One mistake is to suppose that the dominant mechanisms of human energy exchange serve the physiological regulation of body weight or fat content. The other is to regard changes in diet or even the broad lifestyle as a single component of treatment for obesity.

## Stepped weight change from each maintained behaviour change

The thermodynamics of exchange of energy between a physical object and its environment states that a change in body energy content is caused by a difference in rates (MJ per day or week) between input and output. This is contrary to the widespread presumption that a balance between cumulative amounts (MJ) of energy input and/or output determines body weight or fat mass. The change in resting metabolic rate resulting from weight change [7] reduces the difference between energy expenditure and intake rates until weight change comes to asymptote - a step change from baseline.

The introduction of engineering control theory into physiology reinforced the notion that the energy content of a body is a balance of amounts of energy intake and expenditure [8]. Thermostats control the amount of heating or cooling by the machinery in accord with the user’s setting of a pointer on a dial to the desired temperature. No such set point, even an adjustable one, is required by the concept of homeostasis in which Walter Cannon enshrined Claude Bernard’s idea of stabilisation of the internal environment against disturbances from the external environment [9].

## Personal regulation by equilibration including social influences

The behaviour that regulates weight of a human body is primarily influenced these days by personal equilibria among sociocultural norms of physical appearance and the states of markers of healthy living. The currently most influential reference value operative in the controls of human energy intake and expenditure is probably the healthy range of BMI, with recently strengthened advocacy of target waistline [10].

Specific examples of moment-to-moment social influences on ingestion or rated appetite include labelled energy value of a sweetener [11] or fat contents of a food [12, 13], how much of a test food was eaten by other people [14], what was eaten during yesterday’s version of the test meal [15] and a wide variety of other examples, not always recognised as signals from the culture [16].

These cultural influences are part of the overall counterpoise among negative feedback mechanisms in a person’s life. Environmental and physiological factors—i.e., both exteroceptive and interoceptive signals—combine with social factors on the same mental scale of causal strength [11, 17]. Human choices treat influences from the social environment, such as communications on the wrapping of a food item, in the same way as influences from the material environment, such as the appearance, smell and feel of the unwrapped item or the visible closeness of a stairway for descent. During each action, attention integrates a personal selection among social and physical options, transiently equilibrating among signals from sensing, acquired beliefs, emotional reactions and active dispositions [18]. The future challenge for prevention of obesity is to measure the major components of social and physical influence on each pattern of eating, drinking, exercise or thermal comfort as they combine on a usual occasion in each individual in a representative sample of a specified population [19].

First, though, we need to identify the habits that influence weight and measure how much asymptotic loss of weight is contributed by a sustained change in each behaviour pattern. Effective habit changes can then serve as components in a weight loss programme and its RCTs, as detailed below.

## Multiple components of diet and physical activity

The second major culprit for the failure of weight loss programmes to keep weight off in the long term is the single-component model for RCTs. This approach to evidence-based medicine erroneously reduces the scientific method to the comparison of an experimental condition with any sort of control condition that lacks the hypothesised therapeutic element. The fallacy is compounded by bundling multiple components under operationally meaningless terms such as ‘lifestyle’, ‘diet’ and ‘physical activity’, purporting to represent a measurable unity.

Weight-controlling components of a person’s diet are unlikely to be clearly identified while nutrition remains confined to biochemical science. For example, a major focus of current efforts to prevent obesity is reducing the amount of sucrose in the diet. The inclusion of psychological and social sciences in nutrition gave rise to the hypothesis that the most fattening single habit was the consumption of “extras” at the end of or soon after a regular meal [20]. Among children and young people, one of the commonest dietary habits between meals is the drinking of sugar sodas, sometimes accompanied by a packet food item. In adults, prevalent drinks away from meals may be coffee or tea with sugar and/or cream and maybe a cookie, or beer, wine or spirits, perhaps with savoury “nibbles”. The hypothesis that switching to zero-calorie drink breaks makes a worthwhile contribution to lowering weight has yet to be tested by an adequately designed RCT.

The essential components of a weight loss programme are locally recognised habits that contribute to the rate of exchange of energy between the body and the environment—namely, specific patterns of eating, exercise or thermal comfort. Some of these routine practices may be changed, initially at least, by gastric surgery, anti-obesity medication, motivating cognitive-behavioural therapy, dietetic counselling and/or physicians’ and nurses’ encouragement to take more exercise and to eat more fruit and vegetables and fewer snack foods. Hence, the research priority is to measure accurately how much a specified change in a well-known behaviour pattern contributes to weight loss for as long as that change is maintained [6].

## Randomised sequences of behaviour components

Recent developments of design and analysis of clinical trials by social scientists encompass evaluation of the full organisational complexity of delivering a treatment programme, even when it has just a single component [21, 22]. RCTs for programmes that have multiple components compare conditions within a group of participants, instead of assigning each condition to a different group. Components are introduced in succession and the sequences are randomised among participants, not the component conditions.

The simplest version of this design is crossover of conditions, with the first condition providing the baseline for the second [23]. The original version, proposed for behavioural interventions using multiple baselines [24], was later elaborated into complex time series analyses [25]. A pragmatic alternative is sequence-balanced regression from each behaviour change to each outcome. With relatively short series of components, a modest number of participants provides enough data for a robust estimate of the step weight change from a maintained shift in a behaviour pattern. The precision of this estimate is measured by standard statistical parameters such as confidence limits [6].

Firm evidence of the effectiveness of a weight loss programme component depends on three further major improvements: communal identification of potentially effective patterns of behaviour, monitoring change in each behaviour pattern and its persistence or abandonment, and factorial design with single-predictor regression analyses.

## Identification of each pattern of behaviour

The participants in weight loss programmes and trials are the experts on their own distinct patterns of eating, drinking, moving about and keeping warm or cool. Hence, those responsible for offering evidence-based advice should use their clients’ own words, i.e., the consensus on the description of a customary pattern of behaviour in their community. This is key to hard evidence on the realities of weight control [21, 22].

What needs to be communicated in professional delivery of a weight loss programme is therefore the exact behaviour carried out by the participant in ordinary life that has been shown to be effective in reducing weight in the participant’s context. To elicit patterns of behaviour within a locality, small groups experienced in a particular area, guided by an independent convenor, gain a consensus on phrases from diary records [26]. More formally, those living in the everyday situation sort subsets of phrases by judgments of identity [27] or degrees of dissimilarity [28] onto the one statement of a shared practice. Examples in English-speaking countries might include variants of “a full breakfast”, “a light lunch”, “beer with salted peanuts”, “a walk to the shops”, “a run round the park”, “watching a film” or “turning the heating down overnight”. One indicator of the validity of a consensus description is the percentage of the panel who opt for that wording.

## Monitoring behaviour throughout the trial

In order to identify causal direction between changes in behaviour and weight, and to measure the effect of the behaviour change on the outcome, a trialist’s use of a component of the weight loss programme must be monitored before, during and after the intervention. The mechanism of a drug’s action during a trial may not need monitoring because of sufficient evidence from preclinical research. In contrast, evidence on the mechanism of action of behaviour on weight comes from tracking the message conveyed personally in the clinic or broadcast to the public throughout its reception and implementation by each trialist.

Prescription without monitoring compliance is questionable in several ways. Many participants regain lost weight within the year. The simplest explanation is neither metabolic nor mental; rather, trialists do not maintain the changes in habits that cause the initial loss in weight. Established methods of dietary assessment cannot estimate the rate of energy intake at the individual level but physical activity questionnaires are sufficiently realistic for the grouped data to show that the initial increase in exercise has lapsed [29]. Over the subsequent 20 years, there has been no disconfirmation of this entirely behavioural explanation for the general failure of weight loss programmes [30].

The frequency of occurrence of a particular pattern of behaviour is a primary measure of its contribution to the rate of energy intake or expenditure [6]. Changes in size of a portion or a meal may be less influential. In any case, such changes in range of amplitudes of an eating or exercise habit can be analysed as separate changes in frequency of each size range.

Frequency of a repeated behaviour pattern is best measured as the reciprocal of time intervals between successive episodes [6]. Assessments of diet or physical activity concern amounts of foods and beverages or types of movement and stillness, rather than occurrences of discrete patterns of ingestive or locomotor behaviour. Diary records may not state the clock-time of each episode. Direct questions about frequency leave open the possibility that the number given in response is constructed without recall of any actual event [31, 32]. At best the answer may be biased by the time since the most recent occasion. Instead of “how often do you …”, respondents should be asked for specify events; for example, “when did you last do …” and then “when most recently before that?”

The exact frequency of a habit is calculated from the trialist’s record of the timing of each occasion that pattern of behaviour is performed [6]. To influence weight substantially, an ordinary habit has to be repeated at least once a week or so; recall of the timing of an event is highly accurate over such periods [34]. Internal checks are provided by overlapping recalls. Thus, the precise weekly frequency of a habit can readily be monitored throughout a programme or trial, up to the last follow-up.

## Measuring the effect of each changed habit on weight

The parameters of a mechanism are measured by regression from tested levels of the input to observed levels of the output. Well known examples include a dose-response function and a relationship between the levels of a stimulus and a response. The slope of the regression line measures the strength of that influence on that outcome. The intercept measures biases from the context of measurement. The regression coefficient measures reliability of the line, or effect size; it is a statistical parameter and in itself has no mechanistic implications. The regression slope value relates weight loss to maintained change in the specified behaviour pattern, e.g., the asymptotic amount of change in weight resulting from a change in frequency of once per week.

Duration of the step change in weight is likely to vary among habits and extents of frequency change. Existing weight loss programmes induce asymptotic weight changes before about 6 months at the latest [33]. The change in a single piece of behaviour has a modest influence on the rate of intake or expenditure and so the step weight change may take only a few weeks at most [6]. The regression slope increases until the asymptote of weight change is closely approached. Hence the length of time taken by the step weight change can be measured by backtracking the regression period to the end of the slope’s climb to maximum from the start of the change in frequency of the tested behaviour pattern.

## Design of a weight loss programme

The improvements above are not confined to preclinical research. A treatment programme can be designed to collect evidence on the effectiveness of each component at keeping weight down while that habit change persists. Indeed, experiments on potential slimming practices are better run within programmes than in isolation. Monitoring the full range of behaviour that may change protects against confounding. Also, the data can be used to test for statistical interactions that challenge the additive model for combining components.

Whether or not a programme enters a trial, the selection of habits to change should be personally tailored on the evidence to date for both effectiveness on weight loss and feasibility of maintenance. The change in frequency of a habit should be well within the range observed in the sampled population, e.g., between quartiles, or outer deciles at widest, and can be decided by each individual because randomisation in the multiple baseline design is of sequences of behaviour to change, not amounts of change.

The main addition to an adequately designed treatment programme to make it a RCT is the randomisation of participants among sequences of components. Randomisation to an arm of the trial receiving usual care is needed for economic and logistic analyses but not for scientific measurement of the effects of the programme.

Running triallists through a cycle of weight loss and regain for a year (or even less) adds nothing to what we already know about healthy eating or prevention of obesity**-**related disease. Any weight loss programme, not just a trial, should measure incidence of morbidity, disease risk factors, and regain of body weight and fat content in replications of at least two cycles through the seasons (24 months). High rates of drop-out may well arise from habit changes that are unsustainable and therefore should be replaced. Whatever the overall outcomes, the regressions from each component provide evidence from direct and precise measurements of weight change by specific habits that should be used in the design of any subsequent weight loss programme, with or without RCT.

## Conclusion

Improved effectiveness of weight loss programmes is crucial to showing the benefits of avoiding overweight and obesity. Improvements depend on recognising that diet, physical activity and thermoregulation each have multiple behavioural components. Programmes and trials need to specify habits in participants’ own words, monitor changed frequency of each habit throughout a programme and its follow-up and then measure the asymptotic weight loss generated by a change in frequency of once per week.
